# Challenges with implementing malaria rapid diagnostic tests at primary care facilities in a Ghanaian district: a qualitative study

**DOI:** 10.1186/s12936-016-1174-0

**Published:** 2016-02-27

**Authors:** Nana Yaa Boadu, John Amuasi, Daniel Ansong, Edna Einsiedel, Devidas Menon, Stephanie K. Yanow

**Affiliations:** School of Public Health, University of Alberta, Edmonton, Canada; Nursing Best Practices Research Center, School of Nursing, Faculty of Health Sciences, University of Ottawa, Ottawa, ON Canada; Kumasi Collaborative Center for Research in Tropical Medicine, (EOD Group) KNUST, Kumasi, Ghana; Research and Development Unit, Komfo Anokye Teaching Hospital, Kumasi, Ghana; Department of Communication and Culture, University of Calgary, Calgary, AB Canada; Health Technology and Policy Unit, School of Public Health, University of Alberta, Edmonton, Canada; Alberta Provincial Laboratory for Public Health, Edmonton, Canada

**Keywords:** Rapid diagnostic tests (RDTs), Malaria, Guideline compliance, Primary care, Healthcare provider, Health system, Ghana

## Abstract

**Background:**

Rapid diagnostic Tests (RDTs) for malaria enable diagnostic testing at primary care facilities in resource-limited settings, where weak infrastructure limits the use of microscopy. In 2010, Ghana adopted a test-before-treat guideline for malaria, with RDT use promoted to facilitate diagnosis. Yet healthcare practitioners still treat febrile patients without testing, or despite negative malaria test results. Few studies have explored RDT implementation beyond the notions of provider or patient acceptability. The aim of this study was to identify the factors directly influencing malaria RDT implementation at primary care facilities in a Ghanaian district.

**Methods:**

Qualitative interviews, focus groups and direct observations were conducted with 50 providers at six purposively selected primary care facilities in the Atwima–Nwabiagya district. Data were analysed thematically.

**Results:**

RDT implementation was hampered by: (1) healthcare delivery constraints (weak supply chain, limited quality assurance and control, inadequate guideline emphasis, staffing limitations); (2) provider perceptions (entrenched case-management paradigms, limited preparedness for change); (3) social dynamics of care delivery (expected norms of provider-patient interaction, test affordability); and (4) limited provider engagement in policy processes leading to fragmented implementation of health sector reform.

**Conclusion:**

Limited health system capacity, socio-economic, political, and historical factors hampered malaria RDT implementation at primary care facilities in the study district. For effective RDT implementation providers must be: (1) adequately enabled through efficient allocation and management of essential healthcare commodities; (2) appropriately empowered with the requisite knowledge and skill through ongoing, effective professional development; and (3) actively engaged in policy dialogue to demystify socio-political misconceptions that hinder health sector reform policies from improving care delivery. Clear, consistent guideline emphasis, with complementary action to address deep-rooted provider concerns will build their confidence in, and promote uptake of recommended policies, practices, and technology for diagnosing malaria.

## Background

The World Health Organization (WHO) estimates about 214 million cases of malaria occurred worldwide in 2015, leading to 438,000 deaths [1]. Other experts argue that these reports underestimate the actual burden [2]. Almost 90 % of the disease burden occurs in sub-Saharan Africa, where young children below 5 years of age account for more than 78 % of global malaria deaths [3]. Early diagnosis and effective treatment within the first 24 h of symptom onset are vital to prevent complications leading to death from malaria, especially among young children and pregnant women [4].

The complete clinical presentation of malaria is highly variable, making it poorly distinguishable from several other febrile illnesses [5, 6]. Quality-assured microscopy, considered the gold standard for diagnosing malaria, is also difficult to sustain in resource-constrained environments [7]. For decades, febrile illnesses in settings including Ghana have been treated presumptively as malaria. This practice enabled prompt delivery of life-saving treatment to reduce mortality especially among children under 5 years of age [8]. However, wide availability of mostly inexpensive anti-malarials led to rampant presumptive diagnosis (and misdiagnosis) of most fevers as malaria, and inappropriate anti-malarial consumption [9, 10]. Indiscriminate anti-malarial use, including incomplete dosing for repeated malarial infections, is prevalent in high transmission areas [9, 11]. This fosters the emergence and spread of drug resistance [12], exacerbates a vicious cycle of illness and household poverty, and drains limited national and donor resources [13].

The WHO recommends diagnostic testing to confirm malaria before providing anti-malarial treatment to suspected cases [4]. This recommendation aims to limit indiscriminate anti-malarial use, and is premised on three factors: (1) emerging parasitic resistance to anti-malarials; (2) declining malaria transmission in previously high transmission areas; and (3) increased availability of diagnostics, notably, rapid diagnostic tests (RDTs) for malaria in resource-limited environments [14]. RDTs do not require much infrastructure or technical expertise, further questioning the rationale for presumptive treatment in the absence of microscopy [8, 15]. Yet RDT implementation in Ghana and other sub-Saharan African countries remains low. Optimizing RDT use in these settings is vital to expand diagnostic coverage for malaria, while ensuring rational use of anti-malarials [16–18].

Malaria transmission in Ghana is endemic and mostly uninterrupted, with peaks in the rainy seasons. The entire population of over 24 million is at risk of infection year-round [19]. Over three million cases were reported in 2013, accounting for 38 % of all outpatient illnesses and 36 % of hospital admissions. Malaria is also the leading cause of morbidity and mortality among children under 5 years of age, among whom the disease accounted for a third of all deaths in 2013 [20].

Despite adopting the WHO’s test-before-treat recommendation for malaria as national policy in 2009, presumptive treatment practices are widespread across the country. Fewer than 33 % of suspected malaria cases in Ghana were confirmed through parasitological diagnosis in 2013. Rural access to malaria diagnostics remains relatively poor, though these areas bear a disproportionate share of the disease burden [14, 21]. Moreover, where RDTs are used, studies conducted prior to national adoption of the test-before-treat approach found up to 45 % of RDT-negative patients in a Ghanaian setting still received anti-malarial treatment [16, 17]. Evidence from other endemic sub-Saharan African settings suggests between 10 and 80 % of malaria-negative patients are prescribed anti-malarials [22]. This indicates variable and often poor providers’ compliance with the test-before-treat guideline.

Little is known about the factors directly influencing RDT use during routine malaria testing at primary care facilities in endemic settings. The main objective of this study was to determine which factors influenced RDT implementation for routine malaria management at primary care facilities in the study district.

## Methods

This study reports descriptive findings from observations, qualitative interviews and focus groups discussions conducted with 50 primary care providers in the Atwima–Nwabiagya district to elicit their perspectives on RDT use and implementation at their facilities. This data collection formed part of a focused ethnography to identify the determinants of providers’ compliance with the test-before-treat guideline for malaria in the study district. The overall study was guided by a conceptual model that supported the exploration of guideline compliance among primary care providers in the study district. The detailed methodology was published separately [23].

Based on studies of RDT use in similar settings [24–28] and Ghana’s National Malaria Control Programme (NMCP) strategic plan [29], guideline compliance in this study was defined as a two-part commitment from the healthcare provider: (1) to test a patient suspected of having malaria using a RDT (or microscopy where available); and (2) to subsequently manage the case in a manner consistent with the test result.

### The conceptual model

**Fig. 1:**
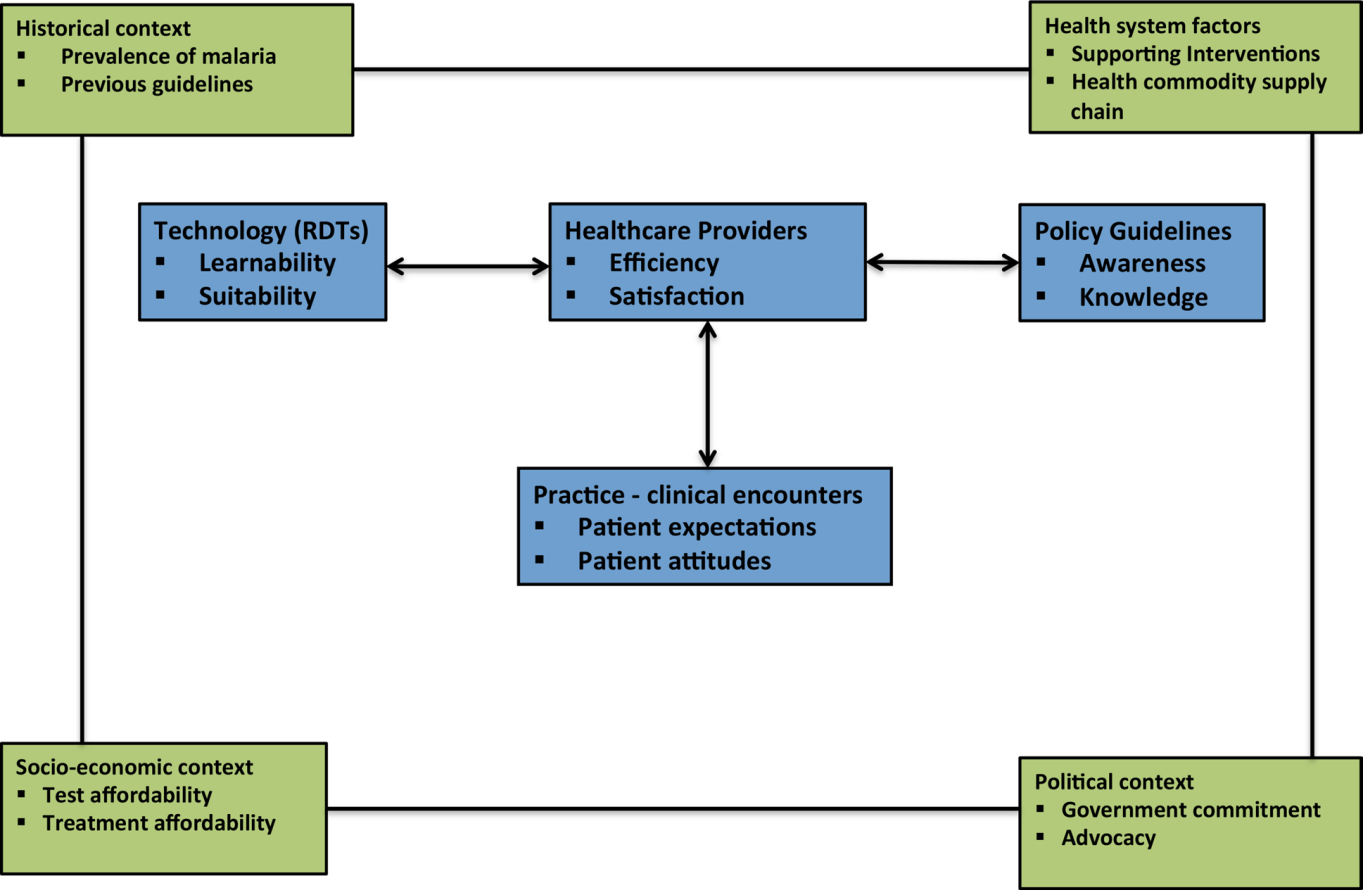
Conceptual framework for investigating healthcare providers’ compliance with the test-before-treat guideline for malaria in a Ghanaian district. The four *outer boxes* in *green*—historical, health system factors, socio-economic factors, and political factors—represent the broader constructs that characterize the context of primary care delivery in the study setting. These contextual factors mediate the processes of RDT uptake for malaria management within that context, and invariably influence providers’ compliance with the test-before-treat guideline for malaria. The four *inner boxes* in *blue* indicate the key interactions that directly influence RDT implementation at primary care settings. The *double-headed arrows* indicate bi-directional influence among these components

The conceptual model (Fig. 1) was informed by the literature on RDT acceptance and use among providers in sub-Saharan African settings [17, 24, 30, 31]. Three key interactions can be identified involving providers and RDTs that affect providers’ compliance with the test-before-treat guideline for malaria. Interactions between: (1) providers and RDTs, expected to affect perceptions of utility, suitability and effectiveness [17, 24, 31]; (2) providers and the policy guideline, considered to influence knowledge, understanding, and application of recommendations for malaria management [32, 33]; and (3) providers and patients—the hub of technology (RDT), policy (test-before-treat) and practice (case management) for malaria, where guideline compliance is demonstrable [34–36]. Moreover, the context of primary care delivery in the setting was expected to influence these interactions [37].

### Sampling and recruitment

The district was selected in consultation with a paediatrician specialist (DA) with extensive research experience in the study area. Malaria was the leading cause of morbidity in the district, with 160,000 reported cases in 2011. Over 40 % of hospital admissions, and 60 % of all-cause mortality among children below 5 years of age in 2011 were attributable to malaria [38]. The Ghana health service (GHS) operated five of the 17 primary healthcare facilities, including four health centres and the district hospital. The remaining 12, including three hospitals, three health centres, one clinic, and five maternity homes, were private, for-profit facilities. Six facilities were purposively selected for provider representativeness, through consultations with a study advisory group, comprising community leaders and key informants familiar with health-seeking patterns in the district. The sample included three pairs of complementary government and private facilities providing services at community, sub-district and district levels respectively (Table 1). Health facility (HF) 1, the smallest, was a private maternity home. The nurse midwife in charge was the main prescriber responsible for clinical decision-making. The private (HF3) and government (HF6) hospitals each had one medical doctor who prescribed together with nurses and at HF 6 with physician assistants. After initial discussions with the district health directorate and heads of selected facilities, 50 providers (Table 2) were directly approached on site to discuss the study and invited to participate, with written consent.

**Table 1:** Characteristics of included study facilities

Generic facility ID	Ownership or operating authority	Facility type	Represented level of primary healthcare delivery	Head of facility (provider cadre)	Available services	Availability and type of malaria diagnostic service—RDT or microscopy	Reasons for limited or no availability of a particular diagnostic service
Health facility (HF) 1	Private	Maternity home	Community	Nurse/midwife	Basic preventive Curative for minor ailments	None	Prolonged district-wide RDT stock-outs including private wholesale/retail suppliers
HF2	Private	Clinic	Sub-district	Medical officer (doctor)	Preventive Limited curative	RDT—limited, irregular supply	Prolonged district-wide RDT stock-outs including private wholesale/retail suppliers
HF3	Private	Hospital	District	Medical officer (doctor)	Curative Limited specialist services E.g., surgery, delivery	Microscopy—routine use	HOF preference backed by infrastructural capacity for quality-assured microscopy
HF4	Government	Health center (small) 40–50 patients per day	Community	Physician assistant	Basic preventive Curative for minor ailments	RDT—limited, irregular supply Microscopy—limited basis	Prolonged district-wide RDT stock-outs Limited recovery on facility costs for reagents needed for microscopy
HF5	Government	Health center (large) 100–200 patients per day	Sub-district	Physician assistant	Preventive Limited curative	RDT—limited supply Microscopy—limited basis	Prolonged district-wide RDT stock-outs Limited recovery on facility costs for reagents needed for microscopy
HF6	Government	District hospital	District	Medical officer (doctor)	Curative Limited specialist services E.g., surgery, caesarean section, delivery	RDT—limited supply and use Microscopy—routine use	Prolonged district-wide RDT stock-outs

**Table 2:** Characteristics of included providers per data collection method at each facility

Study facility	Facility type	Data collection method(s) (number held, if applicable)	Participating provider cadre(s) (number per data collection method)	Total number of participating providers per included study facility
HF 1	Maternity home Private Community level	Non-participant observation 2 h × 5 days = 10 h	Nurse mid-wife (1) Healthcare assistants (2)	4
		Informal interviews (3)	Nurse mid-wife (1) Healthcare assistants (2)	
		Focus group discussion (1)	Nurse mid-wife (1) Healthcare assistants (2) Nurse (1)	
HF 2	Clinic Private Sub-district level	Non-participant observation 3 h × 5 days = 15 h	Medical officer (1) Biostatistician/records officer/testing officer (2)	3
		Informal interview (2)	Medical officer (1) Biostatistician/records officer/testing officer (2)	
		In-depth, semi-structured interview (1)	Medical doctor (1)	
HF 3	Hospital Private District level	Non-participant observation 8 h × 5 days = 40 h	Medical officer (1) Nurses (7) Nurse/midwife (1) Laboratory personnel (3)	12
		Informal interviews (5)	Medical doctor (1) Nurse (1) Lab persons (1)	
		In-depth, semi-structured interview (1)	Health facility administrator	
HF 4	Health center (small) Government Community level	Non-participant observation 3 h × 5 days = 15 h	Physician assistant (1) Laboratory personnel (2) Nurses (2) Health care assistants (1)	6
		Informal interviews (3)	Physician assistant (1) Laboratory personnel (2)	
		In-depth, semi-structured interview (2)	Physician assistant (2)	
HF 5	Health center (large) Government Sub-district level	Non-participant observation 3 h × 5 days = 15 h	Physician Assistant (2) Laboratory Personnel (2) Health extension worker (2)	11
		Informal interviews (3)	Physician assistant (1) Laboratory personnel (2)	
		In-depth, semi-structured interview (4)	Physician assistant (1) Nurse (prescriber) (2)	
		Focus group discussion (1)	Physician assistant (2) Enrolled nurse superintendent (1) Nurse/midwife (1) Health care assistant (1) Dispensing technician (1)	
HF 6		Non-participant observation 5 h × 5 days = 25 h	Medical doctor (1) Nurse (3) Physician assistant (1) Laboratory personnel (4)	14
	Hospital Government District level	Informal interviews (5)	Medical doctor (1) Nurse manager (1) Laboratory personnel (3)	
		In-depth, semi-structured interview (4)	Medical doctor (1) Nurse manager (1)	
		Focus group discussion (1)	Nurse manager (1) Nurses (3) Laboratory personnel (1) Healthcare assistant (1)	
Overall total				50

### Data collection and analysis

Field research was conducted in the study district from November 2011 through October 2012.

#### Direct observation

Participating providers were observed for one week at their facilities during daily clinical encounters with suspected malaria patients. Observations lasted 2–3 h a day at smaller facilities, and 5–8 h at larger facilities with longer periods of steady clinic attendance. In accordance with standard methodology, activities of interest were noted during observations [39], such as providers’ requests for RDTs and their reactions to negative test results. Where necessary, informal interviews helped clarify observed behaviours [40].

#### Interviews

Prescribers were interviewed individually, in English, at their facilities using a topic guide. Interview questions explored providers’ experiences with RDT use, guideline knowledge, and perceived challenges or advantages with implementing the test-before-treat approach.

#### Focus group discussions (FGDs)

A total of three focus group discussions (FGDs) were conducted at a maternity home, a health centre and a hospital, representing each of the included levels of care delivery. Sites were selected based on staff sizes and availability (Table 2). Two were moderated in English, and the third at a remote facility was moderated in the local Akan dialect, to accommodate the participants’ language preference. This group included a co-moderator, a public health nurse well known in the community, and fluent in Akan. Open-ended questions encouraged group interaction, and explored diverse perspectives regarding RDTs and the test-before-treat guideline that were identified during interviews. Audio-recordings and handwritten notes were captured during interviews and FGDs. Digital recordings were translated where necessary, transcribed, and transcripts checked for accuracy. Summary notes prepared after interviews and FGDs were used to assess topic saturation [41]. Transcripts were analysed thematically to address the study objectives using Roper and Shapira’s approach [42]. Participants’ responses were closely examined to uncover any existing patterns by age, gender, facility, or provider type. Participants and local advisors were consulted for further clarification of responses where necessary.

### Ethics

The Health Research Ethics Board at the University of Alberta and the Committee for Health Research Publications and Ethics at the Kwame Nkrumah University of Science and Technology in Kumasi approved the study protocol. All participants provided individual informed consent. Providers also obtained verbal consent from patients and assent from caregivers of minors prior to observations.

## Results

Fifty healthcare providers (HCPs) at six primary care facilities in the study district participated in total. There were no refusals to participate. Key informants identified five health policy and administrative officials at local and national institutions (Table 3) whose insights on systemic challenges to primary care delivery highlighted contextual factors affecting RDT implementation at national and local levels. The main themes identified within the data were limited health system capacity, provider perceptions on malaria and its management, provider-patient interactions, and political factors influencing health sector policy reform.

**Table 3:** Characteristics of representative health administrative/policy officials included in informal interviews (consultative discussions)

Institution or organization	Representative level of administration/policy oversight	Number of consultative discussions held
District (local)	District Health Directorate	2
	District Health Management Team DHMT	1
Regional	Regional Medical Stores	3
	Regional Malaria Control Office	2
National	Society of Private Medical and Dental Practitioners—Ghana SPMDP—Ghana	2
	National Health Insurance Authority	1

### Provider characteristics

Female providers were aged between 21 and 77, and males, 31–65 years. Clinical experience ranged from 17 to 56 years among older providers (≥50 years), to 3–9 years among providers 21–35 years of age (Table 2).

### Health care delivery constraints

Several challenges outlined below hampered RDT implementation and consequently guideline compliance for malaria testing among providers.

#### Limited RDT supply

Both public and private providers revealed during in-depth interviews (IDI) and focus group discussions (FGD) that RDT supplies from the district health directorate to their facilities were often insufficient and sporadic. This challenge was more pronounced at remote facilities solely dependent on government supplies, and a major hindrance to routine malaria testing at all facilities. At the time of the study four facilities had limited RDTs while two had none (Table 1). Stock-outs were common, sometimes lasting several months, making providers hesitant to use limited quantities when available, as illustrated in the quotes below:

*“… The main problem with this rapid diagnostic test kit is the erratic availability…”* (HCP, HF6, IDI)

*“… As you go deeper in the hinterland, the availability of tests also goes down and the rate of blind treatment rises…”* (HCP, HF6, IDI)

*“… Before we got our current supplies I think we were out for almost four months!”* (HCP, HF3, IDI)

Malaria testing at public facilities dependent on government RDT supplies was interrupted due to frequent and prolonged RDT stock outs. Some private providers mentioned purchasing RDTs from independent sources when available. Others abandoned RDT use altogether, citing the economic and technical advantages of microscopy over RDTs, as illustrated below:

*“… When… we ask and they don’t have… we sit and wait until when they have it. That is the problem.”* (Government HCP, HF5, IDI)

*“… We believe in diagnosis and having the diagnostic tools available to do your work and do it well…. all our lab technicians are biomedical scientists… why would I want to go for RDT?”* (Private HCP, HF3 IDI)

*“… When I use… microscopy I am just spending 0.10 GHs per client… for the RDT it’s about GHs 2.00. So I would want to go for the cheaper and most efficient one.”* (Private HCP, HF3, IDI)

#### Poor RDT storage, distribution, and regulation

Sub-optimal storage, distribution and regulatory mechanisms further limited RDT implementation. Frequently delayed distribution and limited storage space at the district office meant RDTs were often left outdoors, exposed to sunlight and other weather conditions. Providers, concerned with compromised test quality due to poor storage, had little confidence in the veracity of test results. Infrequent quality assurance and control visits to facilities by authorities further undermined providers’ willingness to use RDTs. A physician assistant at a health centre stated:

*“… What I have observed is that sometimes they bring them (RDTs) here and they are left with about one month or two months to expire. So they may be giving you fake results.”* (HCP, HF 4, IDI)

#### Heavy caseloads

At facilities with microscopy, providers described time constraints, as well as limited and costly reagents as further barriers to malaria testing. Heavy caseloads resulted in long laboratory wait times that precluded test results informing clinical decision-making in real-time. Testing was often arbitrary, or for detained or admitted patients, who often received treatment before results became available, as illustrated below:

*“You also saw how busy our clinic is… I see about 150* - *200 patients a day… In Africa I cannot get that time…* (to test all suspected cases)*.”* (HCP, IDI, HF5)

*“… The patient load is barring us from doing RDTs in the consulting room. But ideally you should be able to do it in the consulting room.”* (HCP, HF5, IDI)

#### Inconsistent guideline information

Guideline reference material including pictorial job aids were not widely available at facilities to support RDT use, particularly among nurses who often attended to patients outside clinic hours. Where available, job aids were not in high visibility areas, and apparently, not accompanied by the necessary communication to ensure their effectiveness. One pictorial job aid on RDT use was observed at the laboratory at HF 6, the district government hospital. A focus group participant here said:

*“The first time I saw it (RDT) in a poster on the (emergency) ward but I really didn’t know what it was.”* (FGD participant, HF 6)

Moreover, the NMCP guideline did not recommend a specific location for RDT use; neither did it allocate primary responsibility for RDT use at the facility level. Not surprisingly, there was a lack of consensus and clarity among providers regarding who and where to conduct RDTs. RDTs were commonly used at laboratories (where available), or at designated sites at the facilities. A midwife and a healthcare assistant at HF 1 (a private maternity home) tested suspected malaria patients in the consulting room. A records officer/receptionist at HF 2, a private clinic, conducted RDTs in a designated corner of the corridor where patients waited on benches. RDTs were commonly referred to as ‘labs’, as were other diagnostic tests conducted by trained laboratory staff. Consequently, patients incurred standard laboratory fees for RDTs, although the Ministry of Health offered them free of charge. Moreover, some providers who routinely prescribed anti-malarials had never conducted a RDT on a patient, especially at larger facilities with diversified staff roles. This contributed to longer turn around times that precluded test results from informing clinical decision-making in real-time.

### Perceptions of malaria and RDTs

#### Perspectives on RDT implementation

All interviewed providers agreed that the guideline was useful to improve malaria management, and equated RDT use with guideline implementation. RDTs were considered appropriate for basic facilities in remote settings without laboratory infrastructure. Providers at hospitals and larger health centres with laboratories clearly stated their preference for microscopy. Nonetheless, both public and private providers prioritized clinical judgment over a malaria test result based on malaria prevalence in the setting, risks and consequences of delayed treatment as stated below:

*“… Looking at the prevalence… the environment that we live in … the number of mosquitoes around that are biting everybody every time… sometimes not treating it and going only by the lab tests… may present some problems… in a few (negative) cases… I may still go ahead and give… antimalarial thinking it’s not going to cause any harm. But if we hadn’t treated then probably we may run into complications of malaria.”* (HCP, IDI, HF 2)

Younger providers (≤35 years) echoed the opinions of their more experienced colleagues, as captured here:

*“… Some of us have been doing the work for so many years and if you see and if you take very good history… you will be able to arrive at the diagnosis.”* (HCP, 56 years, IDI, government facility)

*“… The symptoms… determine what happens. If the person complains of a bitter taste in the mouth… you have to know that it’s malaria. Even though the test result is negative, you have to treat it as malaria… the guideline says not to do things that way. But the patient’s recovery is really essential.”* (HCP, 21 years, FGD, private facility)

#### Gaps in training on RDT use

Most providers reported receiving some training on RDT use. Formal training was described as organized workshops with incentives including refreshments, travel/transport per diem allowances, and career development credits towards professional licensure, renewal, or promotion. Informal training involved peer-to-peer, on-site learning without similar incentives. Female providers were dissatisfied with what they described as inadequate in-service training and guideline knowledge, which coupled with high staff turn over resulted in few suitably qualified staff to support RDT implementation. Male providers dismissed these issues as characteristic of resource-constrained settings. The quotes below highlight this difference:

*“… Some (providers)… didn’t go for the training therefore they don’t know the benefit of doing the test… and therefore they are not willing to comply… If somebody goes for a workshop… and the person comes into educate or teach others, some people don’t find it serious to adhere to whatever they are being taught by a colleague…”* (Female HCP, IDI, HF6)

*“…Our place, the staff, they are over hundred. I don’t expect everybody to know about RDTs. Even though ideally they should know. Not everybody can go (for training). But the people who are at the helm of affairs, like me who is a prescriber. Like the lab technicians, who deals directly with the RDTs, they are usually the people they identify…* (Male HCP, IDI, HF5)

#### Insufficient guideline knowledge

Providers were generally aware of the test-before-treat guideline for malaria. However, facility heads, staff leads and laboratory personnel demonstrated more clarity of the underlying rationale than their other colleagues. When asked, some nurses, often the first point of contact for patients, cited the presumptive treatment approach, as shown here:

*“… There could be another cause of the signs and symptoms that you might mistakenly consider to be malaria… to be sure, we should confirm whether a suspected case is or is not malaria.”* (Facility head, HF 4, IDI)

*“…When our patients come to the facility…mostly it is with fever. So we check for fever… if it’s above 38* *°C, with other signs… sometimes, we decide to treat for malaria even if we have not checked… that is the guidelines I know…”* (Nurse, HF 5, IDI)

NMCP documents recommended using microscopy where available, to confirm an inconclusive RDT result. Yet a community-level provider highlighted potentially inconsistent training messages:

*“We attended a workshop on RDTs and we were told that medical assistants must have RDTs on hand in the consulting rooms. So after the person comes back from the lab (microscopy), the medical assistant would use the RDT to confirm.”* (HCP, FGD, HF 1)

### Provider-patient interactions

Providers described patients as accepting of RDTs and their capacity to aid diagnosis. However, they also said patients who tested negative for malaria often asked for treatment, preferring not to prolong the symptoms, or to face additional costs of further testing. Nonetheless, long clinic hours on busy days were a disincentive to routine malaria testing. Tuesdays were designated market days in the study district during which residents engaged in livelihood activities earlier in the day. The influx of patients after midday contributed to high caseloads and prolonged laboratory wait times. Under such pressure, providers resorted to presumptive treatment to enable timely care delivery.

Within this peri-urban district, the catchment area for most facilities included many rural communities. Providers were well acquainted with conditions in their communities, and unwilling to burden patients with the cost of a diagnostic test in addition to potentially inevitable treatment costs for malaria. Furthermore, limited RDT supplies were insufficient to test every suspected case. Presumptive treatment therefore relieved the added pressure of allaying misconceptions of discriminatory case management among patients who weren’t tested.

### Political influences on health sector reform

Key informants from relevant institutions including the national health insurance authority (NHIA), and the Society of Private Medical and Dental Practitioners (Table 4), threw light on the complexity of perceived political influences at the regional level that hampered RDT implementation. Health sector reform policies by the incumbent government aimed at eliminating coverage and reimbursement bottlenecks, fraud, and wastage were considered politically instigated punitive measures, and unpopular among the general populace. Moreover, most providers expressed dissatisfaction with a regional NHIA pilot scheme of capitated fee reimbursements. Government reimbursements to providers were frequently 6–8 months in arrears, prompting providers to apply fees, or to refuse service delivery to insured patients. While national health insurance coverage included free malaria testing using RDTs, other lab tests including microscopy were not covered. Given that RDTs were mostly in short supply, often conducted in the laboratory, and that reimbursements were not forthcoming, insured patients were unknowingly charged for them. Providers also preferred using microscopy to limit costs to their facilities from delayed reimbursements. One prescriber stated:

*“I know they (RDTs) are supposed to be free. But, the various heads of institutions that we are under, they’ve made it that they (patients) pay.”* (HCP, IDI, HF 4)

**Table 4:** Socio-demographic characteristics of interviewed healthcare providers including heads of facilities (HOFs)

Health facility (HF)	Healthcare provider qualification	Gender	Age (years)	Years of practice experience	Years of practice at current facility
HF 1 Maternity home Private Community level	Nurse mid-wife (HOF)	Female	77	56	16
	Healthcare assistant	Male	25	4	4
	Healthcare assistant	Female	21	3	1
HF 2 Clinic Private Sub-district level	Medical doctor (HOF)	Male	65	38	29
HF 3 Hospital Private District level	Medical doctor (HOF)	Male	35	9	2.5
	Health facility administrator (HOF)	Female	34	9	2.5
HF 4 Health center (small) Government Community level	Physician assistant (HOF)	Male	51	14	5
	Physician assistant	Male	31	7	2
HF 5 Health center (large) Government Community level	Physician assistant (HOF)	Male	56	24	18
	Nurse	Female	32	9	7
	Nurse	Male	32	4	2.5
HF 6 Hospital Government District level	Medical doctor (HOF)	Male	51	28	1
	Nurse manager	Female	55	17	1

## Discussion

The general consensus among providers that RDTs are useful indicates potential endorsement of the shift from presumptive to test-based malaria management. Nonetheless, the priority given clinical judgment suggests limited confidence in RDTs. Previous research linked this finding to concerns over the safety of withholding treatment from febrile patients [43]. Clear communication and consistent emphasis of the test-before-treat guideline and its underlying rationale are critical to address deep-rooted provider concerns and to build confidence in the reliability of RDTs. Common complaints of sporadic and inadequate RDT supplies imply systemic health resource availability and allocation constraints beyond the facility level. This finding, supported by previous research in low-income, malaria-endemic settings [44, 45], highlights the critical nature of resource availability and management to RDT implementation.

Limited health system capacity often indicates competing development priorities, and reflects the broader socio-economic and political contexts of primary care delivery in the setting [46]. Given the public health significance of malaria locally and nationally, the 1 % district budget allocation to its management is inadequate. An upward revision should accommodate guideline communication and emphasis, and logistical support for RDT distribution, storage and use.

National dissemination efforts may have been somewhat successful, as providers were generally aware of the guideline. However, the lack of clarity among non-lead and non-laboratory staff suggests gaps in training coverage. Given the multi-factorial influences on training message uptake in low- and middle-income countries, evidence linking investments in health worker training on improved health outcomes is sparse [47]. Widely used approaches that may help to overcome guideline implementation barriers include training, print material and self-audit [48, 49]. Socially innovative initiatives that employ the seniority and experience of long-serving staff may support best practices uptake among new or less experienced colleagues. To better support the needed paradigm shift, programme and policy officials should engage providers in discussing facility-based evidence aggregated at district and regional levels. Such discussions can highlight the growing incidence of non-malarial febrile illnesses, advantages of RDT-supported diagnosis, and anti-malarial resistance development. This will promote buy-in and can inform behavior change communication strategies targeting providers.

Female participants’ dissatisfaction with existing training opportunities suggests potentially gender-related inequities with training coverage. This gap may stem from socio-cultural norms in Ghanaian society, where males enjoy preferential educational and career advancement opportunities. Societal expectations are that females should take a more nurturing role, in and outside the home. Most nurses in this study were female, while physicians, physician assistants, lead staff, and laboratory personnel were generally male (Table 2). Given the socio-cultural and gendered representation among different provider types, planning for in-service training should pro-actively accommodate gender- and provider-based equality. Rotational training opportunities should target nurses, often the first point of contact during and outside regular clinic hours. This will mitigate knowledge, skill and proficiency gaps that limit RDT implementation. As an example, a survey conducted in Sudan found that 76 % of providers nation-wide had not received training on RDT use and less than 50 % had ever received any training through the Sudanese National Malaria Control Programme [50]. The cascade (trainer of trainees) system prevalent in low-income settings relies largely on peer-to-peer knowledge transfer. For this to be effective, periodic review using relevant indicators is essential to identify and eliminate inefficiencies, and to promote accountability. Inadequate staffing levels at peripheral facilities in remote sub-Saharan settings imply that healthcare personnel routinely provide services beyond their professional designation, qualification, or job description [44]. High staff turnover alluded to by participating providers in this study might suggest remaining staff were similarly overstretched. Forecasting for high-volume clinic periods will enable RDT use to facilitate rather than hamper timely clinical decision-making for malaria.

## Limitations

Data generated from this study were based on interview (reported) accounts of experiences with RDT use for malaria management. Triangulation involving focus groups and direct observations was used to corroborate the information, in addition to reviewing relevant government and local documents. These included the NMCP, strategic plan for malaria control [29], guidelines for malaria management and district RDT distribution records. It is possible that providers may have behaved differently while being observed. However, the rationale for their routine behavior of treating fever patients presumptively was to prevent avoidable complications or death. This made it unlikely for providers to behave differently while being observed, given patients would still face the same risks.

## Conclusion

Health system capacity is integral to successful malaria RDT implementation. To maximize the potential contribution of RDTs, providers should be equipped with the requisite knowledge, skill, and resources to accurately diagnose malaria, while accommodating differences in capacity and context across primary care settings. A number of recommendations to address these gaps emerge from this work. (1) Strategic resource planning should support RDT implementation at basic facilities while strengthening laboratory capacity in hospitals. (2) Policy makers should enable equitable in-service training opportunities, and engage providers in policy development for malaria. (3) Policy-practice dialogues should consider the multifactorial nature of the desired paradigm shift for providers, and address topical issues including trends in prevalence of malaria and non-malarial fevers, and anti-malarial resistance development. (4) Experienced providers should mentor junior colleagues to reinforce updated clinical standards. (5) Peer and patient interactions influencing RDT acceptability and use among providers in different primary care settings should be assessed to inform behavior change communication strategies. Collectively, these actions will progressively mediate negative perceptions of RDT use and sustain test-based diagnosis of malaria at all levels of primary care.

## Abbreviations

ACT: artemisinin-based combination therapy
FGD: focus group discussion
HCP: healthcare provider
HOF: head of facility
IDI: in-depth interview
NHIA: National Health Insurance Authority
NMCP: National Malaria Control Programme
RDT: rapid diagnostic tests
